# Benefits and Limitations of a Community-Engaged Emergency Referral System in a Remote, Impoverished Setting of Northern Ghana

**DOI:** 10.9745/GHSP-D-16-00253

**Published:** 2016-12-23

**Authors:** Sneha Patel, John Koku Awoonor-Williams, Rofina Asuru, Christopher B Boyer, Janet Awopole Yepakeh Tiah, Mallory C Sheff, Margaret L Schmitt, Robert Alirigia, Elizabeth F Jackson, James F Phillips

**Affiliations:** a New York City Department of Health and Mental Hygiene, New York, NY, USA.; b University of Basel, Swiss Tropical and Public Health Institute, and Ghana Health Service, Accra, Ghana.; c Innovations for Poverty Action, New York, NY, USA.; d University Research Corporation, Accra, Ghana.; e Columbia University Mailman School of Public Health, New York, NY, USA.; f Jhpiego Ghana, Accra, Ghana.

## Abstract

Although Ghana has a well-organized primary health care system, it lacks policies and guidelines for developing or providing emergency referral services. In 2012, an emergency referral pilot—the Sustainable Emergency Referral Care (SERC) initiative—was launched by the Ghana Health Service in collaboration with community stakeholders and health workers in one subdistrict of the Upper East Region where approximately 20,000 people reside. The pilot program was scaled up in 2013 to a 3-district (12-subdistrict) plausibility trial that served a population of approximately 184,000 over 2 years from 2013 to 2015. The SERC initiative was fielded as a component of a 6-year health systems strengthening and capacity-building project known as the Ghana Essential Health Intervention Program. Implementation research using mixed methods, including quantitative analysis of key process and health indicators over time in the 12 intervention subdistricts compared with comparison districts, a survey of health workers, and qualitative systems appraisal with community members, provided data on effectiveness of the system as well as operational challenges and potential solutions. Monitoring data show that community exposure to SERC was associated with an increased volume of emergency referrals, diminished reliance on primary care facilities not staffed or equipped to provide surgical care, and increased caseloads at facilities capable of providing appropriate acute care (i.e., district hospitals). Community members strongly endorsed the program and expressed appreciation for the service. Low rates of adherence to some care protocols were noted: referring facilities often failed to alert receiving facilities of incoming patients, not all patients transported were accompanied by a health worker, and receiving facilities commonly failed to provide patient outcome feedback to the referring facility. Yet in areas where SERC worked to bypass substandard points of care, overall facility-based maternal mortality as well as accident-related deaths decreased relative to levels observed in facilities located in comparison areas.

## Abstract (precis)

A low-cost emergency and communication transportation system used 3-wheeled motorcycles driven by trained community volunteers. Delivery referrals were redirected from health centers to hospitals capable of advanced services including cesarean deliveries, which was associated with reduced facility-based maternal mortality.

## INTRODUCTION

African nations achieved considerable progress in child health during the Millennium Development Goal era. Despite this progress, maternal and perinatal mortality remain among the leading causes of death throughout Africa. According to the World Health Organization, approximately 800 women die from pregnancy or childbirth-related complications every day.^1^ Nearly 99% of these deaths occur in developing countries and over half occur in sub-Saharan Africa, where only 7% of the global population resides. Most maternal deaths could be prevented if women received timely care when emergencies arise from associated causes, such as hemorrhaging, unsafe abortions, obstructed labor, infection, or eclampsia.^2^

Most maternal deaths could be prevented if women received timely care during medical emergencies.

Nearly all maternal deaths are accompanied by associated neonatal deaths. Although most neonatal deaths are preventable if skilled attendants assist during deliveries,^3^^,^^4^ rates remain high even where child health and survival are otherwise improving. Yet evidence repeatedly shows that facility delivery and appropriate support for newborn care can reduce neonatal mortality if referral services are functioning and attendants are skilled in recognizing problems and immediately providing post-delivery interventions such as “Kangaroo Mother care,” asphyxia management, care for febrile illness, and tetanus prevention.^5^^–^^8^

Public health systems in Africa are therefore making the development of emergency care systems a priority.^6^^,^^7^ The World Health Organization defines 3 core components of emergency care: care provided in the community, during transportation, and at the health facility.^8^^,^^9^ Each component incurs corresponding sources of risk that elevate death and disability: delays in (1) seeking care,^10^ (2) reaching care,^11^^,^^12^ and (3) receiving care upon arrival at the referral facility.^13^ In rural Ghana, and elsewhere in Africa, these delays are driven, respectively, by (1) lack of awareness of the importance of emergency care,^14^ lack of family resources to cover referral costs,^15^^,^^16^ and concerns about the quality of care^17^; (2) poor road conditions,^18^ a scarcity of vehicles,^19^^,^^20^ and limited means of communication^21^; and (3) inaccessibility of competent providers of essential acute care.^20^^–^^22^

Public health systems in Africa are making development of emergency care systems a priority.

While Ghana has a well-organized, decentralized primary health care system, the country has yet to develop clear emergency referral service guidelines. The Upper East Region is one of Ghana's most impoverished and remote localities: The 13 districts of the region are characterized by a scarcity of vehicles, poor road networks, impassible terrain, and geographic barriers to reaching health services.^23^^,^^24^ Patients in urgent need of acute care reach health facilities by walking or riding bicycles, donkey carts, or motorbikes. In all districts of the Upper East Region, ambulances are typically absent, in disrepair, or located so remotely from communities that they fail to address emergency needs. Even where equipment is available, there is no organized emergency communication system to link one level of care to another and ensure that referrals are successfully executed. Cultural norms can also constrain timely care seeking behavior. Moreover, since Ghana's National Health Insurance Scheme does not cover costs associated with emergency transportation, referral can be prohibitively expensive, with costs further increasing people's hesitation to seek acute care.

In Ghana's Upper East Region, ambulances are absent, in disrepair, or located so remotely that they fail to address emergency needs.

The Ghana Essential Health Intervention Programme (GEHIP) is a systems-strengthening initiative that was designed to increase universal access to health care.^25^ GEHIP's aims are to expand coverage of the national primary health care system with the Community-Based Health Planning and Services (CHPS) initiative at the community level,^26^ and to identify gaps in care for newborns, children, and pregnant women at multiple levels of the health system. In addition to addressing issues that had hindered CHPS scale-up, GEHIP has trained midwives in neonatal resuscitation, provided frontline CHPS community nurses with skills in emergency delivery, and trained CHPS community nurses and community volunteers in community-based newborn care.^25^

GEHIP also documented the urgent need for emergency referral services, including emergency obstetric care, in the Upper East Region. In response, the Ghana Health Service (GHS) pilot tested an emergency referral program, the Sustainable Emergency Referral Care (SERC) initiative, for all types of medical emergencies. This article provides a summary of the initiative components and evaluates the effectiveness of the program using results from mixed-methods implementation research.

## THE SERC INITIATIVE

The SERC initiative aimed to develop a community- and subdistrict-level emergency referral system that would improve survival in impoverished rural Ghanaian communities. To address common access, organizational, and knowledge barriers to emergency care services, SERC was designed as a low-cost emergency transportation and communication system together with community education activities. The program aimed to facilitate rapid transport of patients from their community locations or subdistrict health center to higher levels of care.

SERC was designed to address barriers to emergency care services through a low-cost system of transportation, communication, and education.

GEHIP used the tools and methods of participatory planning^27^^–^^29^ to design and implement SERC in collaboration with community members as well as community, subdistrict, district, and regional health system officials. GEHIP research staff held meetings, focus group discussions, and in-depth interviews with community members, frontline workers, and supervisors throughout the planning process to solicit stakeholder advice. Project research assistants were recent graduates of local universities who were hired by GEHIP and assigned to each District Health Management Team to support SERC implementation activities and liaise across levels of the health system.

**Figure:**
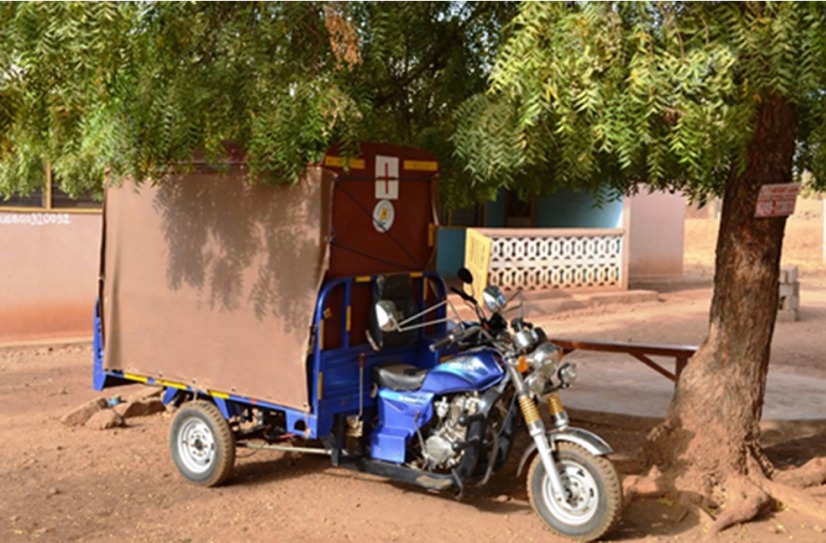
Three-wheeled motorcycles, known as Motorkings, served as emergency transport vehicles in the pilot districts of northern Ghana. Structural modifications were made to enhance patient safety and comfort, including extended reviewmirrors and a tarpaulin to provide privacy and protection.

SERC was conducted initially as a 5-month pilot program with community stakeholders and health workers in a subdistrict of Bongo District in 2012. In July 2013, the program added 12 subdistricts of the Upper East's Bongo, Builsa North, and Builsa South districts for a trial that served a population of approximately 184,000. Remaining districts of the Upper East Region, where social, economic, and ecological conditions are comparable to the SERC coverage areas, served as comparison areas for evaluating the program. The SERC interventions included a referral strategy informed by an assessment of population needs and health systems capabilities; adequately resourced referral centers; active collaboration between referral levels and across sectors; formalized communication and transportation arrangements, with specific protocols specified for referrer and receiver and mechanisms for ensuring supervision and accountability; affordable service costs; capacity to monitor effectiveness; and policy support.

SERC was developed as a component of the health systems development program GEHIP, and SERC scale-up was led by GEHIP staff based at the Upper East Regional Health Directorate (RHD).

### Transportation

For the expanded pilot study that began in 2013, GHS procured a fleet of 3-wheeled motorcycles known as Motorkings to serve as emergency transport vehicles. The 24 SERC Motorkings were distributed among 12 subdistricts of Bongo, Builsa North, and Builsa South districts in the Upper East Region. Based on driver advice from SERC's pilot phase, structural modifications were made to the Motorkings to enhance patient safety and comfort. These adjustments involved installing a welded frame and tarpaulin to provide privacy and protection for patients during transport, extended rearview mirrors for maximum visibility, a mattress and safety belt for patients, a seat for an accompanying health worker, and a hook for intravenous drips. To identify the Motorking vehicles as ambulances, each was marked with the GHS logo and a red cross. Each vehicle was equipped with a first aid kit, a spare tire and jack, and protective rain gear for drivers. Recognizing the importance of vehicle maintenance, vehicles were routinely serviced by staff mechanics from the Upper East RHD. Spare parts were procured and kept in stock at the RHD to ensure timely repair in the event of breakdowns.

GHS purchased and modified 24 three-wheeled Motorking motorcycles for use as ambulances.

Geographic information systems (GIS) data were used to estimate the optimal placement of ambulance stations and configuration of catchment areas to ensure community access to an ambulance.^28^ The SERC ambulances were deployed to 9 subdistrict health centers, 12 community health posts, and 3 communities that lacked facilities or community resident nurses. In Ghana, community health posts function as the first point of care, but only half of the planned locations for these facilities are functional. In the 3 locations that lacked health facilities, community leaders were engaged to determine an appropriate location for the community-based ambulance station. In each of these 3 villages, the community chose an assemblyman's or subchief's home as the station due to its centrality, relative security, and social acceptability for this responsibility. The number of Motorkings was based on an appraisal of the equipment required to effectively cover all communities of the 12 subdistricts. The size of the study area, in turn, was determined by the volume of referrals that would be required to provide statistical power for evaluation.

The community selected 48 volunteers (2 per ambulance), who were trained to serve as drivers. Drivers varied in age, but were typically literate young adult men. A collaboration of the RHD's Transport Unit, the Motorking vendor, the Driver and Vehicle Licensing Authority, and the Ghana Red Cross provided training to all drivers in basic first aid, infection prevention, defensive driving, basic maintenance, transport policies, communications protocols, and recordkeeping.

### Communication

Before SERC, no integrated emergency communication system had been established to link patients to emergency care services at the community and subdistrict levels. Therefore, the RHD procured communication equipment: dual-SIM mobile phones were distributed to health facilities, health workers, and volunteer drivers. Emergency phones were assigned to nurses called community health officers who were based at community facilities, in subdistrict health centers, or in district hospitals' outpatient departments. In communities that lacked a resident nurse, a volunteer was provided an emergency phone and cell phone time for calls to emergency numbers. This collaboration with a cell phone vendor ensured that every community had access to a mobile phone for eliciting rapid referral. At the tertiary referral point, the regional hospital designated a phone line in each ward for receiving incoming calls about impending cases.

The regional hospital dedicated phone lines to receiving calls about incoming emergencies.

### Community Engagement

Ghanaian cultural groups have well-defined systems of social organization and community consensus building that rely upon *durbars*, which are open forums for discussing matters of collective importance to the community. In concert with these traditions, the SERC program convened *durbars* in all ambulance catchment areas to explain the intended use of the ambulance, introduce the local SERC health workers and volunteers and review their roles, and discuss the importance of seeking care during emergencies. An emergency phone number was provided to each community and placed on posters at the nearby health facilities and community gathering points. Participating health workers also liaised closely with traditional chiefs and elders, whose support was essential.

GEHIP equipped SERC staff and volunteers with emergency phones and trained them in mobile phone use, criteria for ambulance use, protocols, and recordkeeping. GEHIP staff held quarterly refresher training sessions to ensure that knowledge and skills were retained, and trained district- and subdistrict-level supervisors to oversee SERC activities and provide routine monitoring and supervision. Monthly review meetings were held across worker tiers to discuss challenges that arose and system improvements needed.

Protocols specified various emergency scenarios in the community and facility setting. Key guidelines included verification of emergency by a health worker and alerts to facilities to prepare for incoming patients and minimize delays. Frontline workers at community health posts were trained in basic triage procedures. All patients being transported were to be accompanied by a health worker. Facilities that received an emergency case were required to provide feedback to the referring facility upon discharge to facilitate follow-up scheduling. The program design included routine monitoring of resources and supplies to assess availability of human resources, equipment, medication, and forms.

GHS supported the operating costs of the SERC emergency referral system. Pregnant women and children under 5 years of age were provided free emergency transport. To encourage facility-based delivery, normal labor cases were transported free of charge. Other ambulance users were charged a nominal cost recovery fee (US$2.50–$5.00) that was determined by each District Health Management Team. In one district, the district assembly covered maternal and child referral fuel costs.

Pregnant women and children under 5 years of age were provided free emergency transport.

Health worker feedback was solicited on SERC to continually inform strategies for educating communities about emergencies. Qualitative appraisal methods were used to determine what community members needed to learn regarding emergencies and to identify strategies for developing a culturally appropriate community education program. The aim was to increase capacity in the community to recognize signs and symptoms of emergencies, encourage prompt decision making to seek care, and increase use of SERC. Opinion leaders and community members contributed to the development of educational materials, which were translated into local languages. These materials included educational flip charts (for use by health workers) and informative songs played on local radio stations and on speaker systems in outpatient hospital wards. Dramas depicting emergency scenarios were developed, filmed, and shown at evening durbars, and posters displayed in health facilities and meeting points depicted actions to be taken in emergency situations.^14^ Discussions of the possible harm to SERC that could arise if equipment was misused were integrated into community education sessions.

## METHODS

An iterative systems development approach was employed to continuously refine the SERC initiative in response to community reactions and administrative realities. GEHIP staff and consultants conducted implementation research to identify operational challenges and potential solutions. Methods included a quantitative analysis of key process and health indicators over time, a survey of health workers, and continuous qualitative systems appraisal with frontline workers and community members.

### Time Series Analysis of Key Indicators

Volunteers, health workers, and district supervisors completed monthly SERC monitoring records and submitted them to GEHIP staff at the RHD, who created visualizations of results to help supervisors assess both referral volume by location and the types of cases associated with referral operations. Monitoring included station-specific information on distances traveled, transit times, adherence to protocols, types of emergency, and patient outcomes. The monitoring used technology designed to integrate SERC monitoring into the routine GHS data system operations known as the District Health Information Management System (DHIMS). Educational aids and training sessions were developed to help regional and district-level managers use the DHIMS database for practical decision making.

Monitoring information included distances traveled, travel times, types of emergency, and patient outcomes.

DHIMS data are aggregated at the facility level and provide indicators of the monthly care caseload by indicator and by type of facility (e.g., CHPS, subdistrict health center, or hospital). Monthly DHIMS data are routinely available for all primary health care service points in Ghana, including the community health posts and community nurses involved in SERC, and we used these aggregated data to support the program evaluation. For the purpose of this analysis, the 12 SERC-exposed “treatment” subdistricts provided a basis for assessing the effect of the program. District facilities in the Upper West Region and the remaining subdistricts of the Upper East Region served as comparison areas. The dependent variable was monthly case volume of each relevant indicator; exposure versus nonexposure to SERC in the facility catchment area was the key independent variable.

For the SERC evaluation, we compared DHIMS time series data from SERC facilities with data from facilities located in unexposed districts of the Upper East and Upper West regions. The comparison applied generalized linear mixed models with an exchangeable covariance structure to account for repeated observations.^30^ This basis for inference ensures simultaneous adjustment for autoregressive error in time series models^31^ and hierarchical adjustment formultilevel clustering.^32^ For each dependent variable of interest, a model of monthly time series data takes the form:

$$y_{ij}=\beta_{0}+\beta_{1}x_{ij}+\beta_{2}t_{ij}+\beta_{3}x_{ij}t_{ij}+u_{j}+\varepsilon_{ij}$$

where

*y_ij_* is a DHIMS-reported value of outcome *y* from facility *j* at time *i*,
*x_ij_* is a dummy variable defining whether facility *j* is in the SERC area or in a control area,
*t_ij_* is a dummy variable defining whether time *i* is before or after the start of the SERC intervention,
*u_j_* is a random intercept for facility *j*, and
*ε_ij_* is a random error term for facility *j* at time *i*.

The parameters β_0_, β_1_, β_2_, and β_3_ are estimated by maximum likelihood, with β_3_ estimating the difference-in-difference association of SERC exposure with the number of events of interest recorded by 14 hospital facilities over 70 months of observation. This approach to evaluation represents a regression extension of the Heckman procedure for estimating the impact of interventions in nonexperimental designs.^33^^,^^34^

The sign of the β_3_ coefficient in the equation indicates the direction of the net change in expected monthly case volume between treatment and control: A positive sign implies a positive SERC effect on case volume (i.e., an increase in the treatment facility volume relative to the control), and a negative sign implies a negative SERC effect on case volume (i.e., a decrease in the treatment facility volume relative to the control). For example, a value of +6 for β_3_ in the equation for an analysis of facility-based delivery would indicate that the expected mean number of monthly deliveries in the treatment facilities increased by 6 deliveries relative to the mean volume of deliveries in the comparison area between the pre- and post-intervention periods. Statistical tests for this coefficient assess whether this relative change is significant. We employed a similar difference-in-difference approach to evaluate the effect of SERC on maternal mortality; however, we substituted a generalized linear Poisson model to properly estimate the maternal mortality ratio. Repeated observations within a facility were adjusted by assuming an exchangeable correlation structure.^35^^,^^36^ Table 1 reports robust standard errors obtained via the sandwich operator.^37^ Although the time series models in this analysis have employed conventional adjustments and statistical safeguards, all such models incur an element of instability. Results therefore merit further investigation and validation.^38^

**TABLE 1.** Difference-in-Differences Estimates of the Impact of the SERC Initiative on Hospital-Based Health Measures, Upper East and Upper West Regions, Ghana 2009–2015

	(1)	(2)	(3)	(4)	(5)	(6)	(7)	(8)	(9)
	Deliveries	Cesarean Delivery Rate	Referrals In	Referrals Out	Pneumonia	Other Upper Respiratory Tract	Septicemia	Accidents	Diarrheal Diseases
Treatment area	−52.93*	−0.00651	−12.08**	−6.499	−4.537	−100.5**	2.555	−15.35	20.96
	(26.18)	(0.0113)	(4.345)	(3.735)	(8.015)	(37.8)	(25.98)	(8.088)	(36.05)
Time period	32.55***	0.025**	−3.05*	−6.80***	0.687	35.57	23.64*	−3.24	18.12
	(9.55)	(0.00964)	(1.435)	(1.87)	(12.75)	(47.67)	(11.9)	(3.209)	(19.13)
SERC^a^	−4.88	0.0035	12.27*	1.60	10.99	22.57	35.09	20.52*	11.71
	(12.76)	(0.015)	(5.18)	(3.52)	(12.92)	(49.82)	(41.91)	(9.90)	(33.54)
Constant	89.73***	0.12***	17.45***	12.18***	46.96***	237.40***	28.38	28.17***	72.04***
	(23.81)	(0.02)	(4.17)	(3.04)	(14.13)	(58.81)	(30.88)	(6.84)	(14.96)
Observations	861	795	361	500	787	748	237	796	804
Number of hospitals	14	13	13	14	14	14	10	14	14

Abbreviation: SERC, Sustainable Emergency Referral Care.

Note: Estimates are from multilevel linear regressions of outcomes from monthly hospital records in the Upper East and Upper West Regions of Ghana. Regressions include random facility intercepts to account for clustering at the facility level. Standard errors are calculated assuming an exchangeable correlation structure and are reported in parentheses.

* *P*<.05; ** *P*<.01; *** *P*<.001.

^a^ The SERC effect (difference-in-difference) is given by the interaction of treatment area with time period.

### Survey of Health Workers

A survey was administered to health workers by trained professional interviewers in December 2013 to assess their perspectives on SERC components and challenges. The questionnaire was pretested and then revised based on feedback provided. The sampling frame comprised all staff based at subdistrict- and community-level health facilities that were involved in the SERC program, including staff affiliated with ambulance stations, responsible for referral operations at ambulance stations, or charged with receiving SERC referrals. This yielded a list of 124 health workers and a response rate of 89% (N = 110), as 14 potential respondents were on annual leave during the week the survey was administered. During that 1-week period, the 110 respondents completed the survey instruments as self-administered questionnaires. Respondents were encouraged to provide candid feedback, and were provided with de-identified forms and blank envelopes to preserve anonymity. These procedures assured respondents of confidentiality so that they could answer questions without risk that critical comments would incur supervisory concern or reprisal.

### Qualitative Systems Appraisal

Trained professional facilitators conducted a qualitative systems appraisal in March 2014 to assess community stakeholder, patient, and volunteer experiences with SERC, employing qualitative research procedures that have been applied to CHPS assessment in the past.^28^^,^^29^ An analysis of focus group discussions (FGDs) and in-depth interviews (IDIs) with community stakeholders assessed the acceptability of the intervention at the community level. IDIs were also used to examine patient experiences with SERC, satisfaction with care, and suggestions for improvement. A total of 16 FGDs were conducted with men, women, drivers, and volunteers from the subdistricts implementing the SERC program. Twenty-three IDIs were conducted with chiefs, emergency referral users, and volunteers equipped with emergency phones. To enhance representativeness, each focus group category (i.e., women, men, community health volunteers) was sampled in a different community, and each FGD was held within the community to promote discussion. Respondents provided written consent; all IDIs and FGDs were conducted in local languages Buili and Guruni; and all interviews were tape-recorded, transcribed, and analyzed using the NVivo 9 software package.^39^

## RESULTS

### Findings From the Time Series Analysis of Key Indicators

**FIGURE 1:**
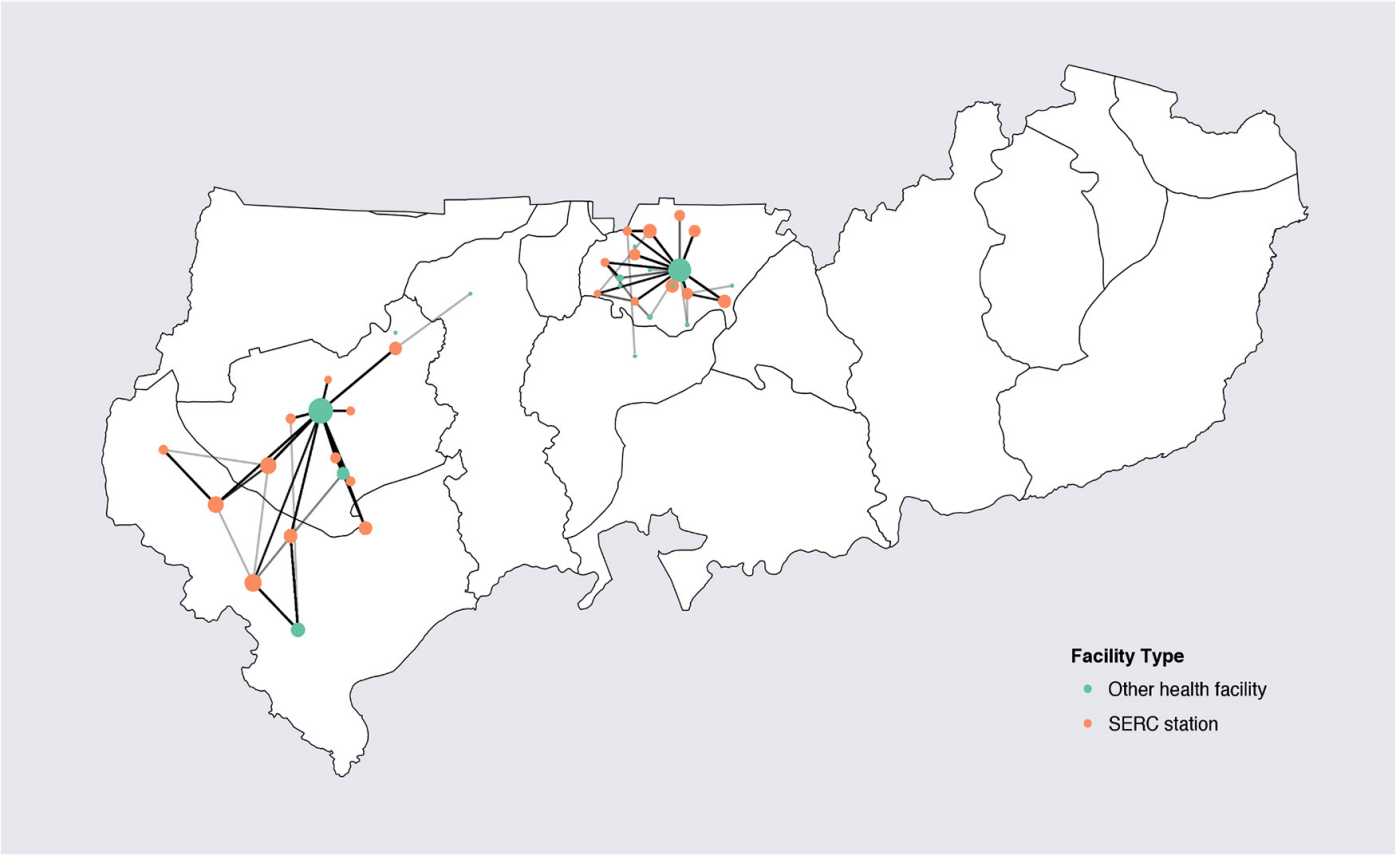
Transportation Routes of Patients Using SERC Services, July 2013–June 2015 Abbreviation: SERC, Sustainable Emergency Referral Care.

From July 2013 through June 2015, 1,290 patients used SERC transport services. The average trip time and distance traveled were 56.6 minutes and 9.92 kilometers, respectively. Most referrals were to higher levels of care at subdistrict health centers and district hospitals, with a high concentration of care occurring at 2 facilities that are well staffed and equipped to manage emergencies (Figure 1). The next most common reasons for referral included malaria, anemia, diarrhea, acute respiratory illnesses, and injury. Most patients (98%) were treated and discharged successfully, while 2% of all emergency referrals resulted in death.

**FIGURE 2:**
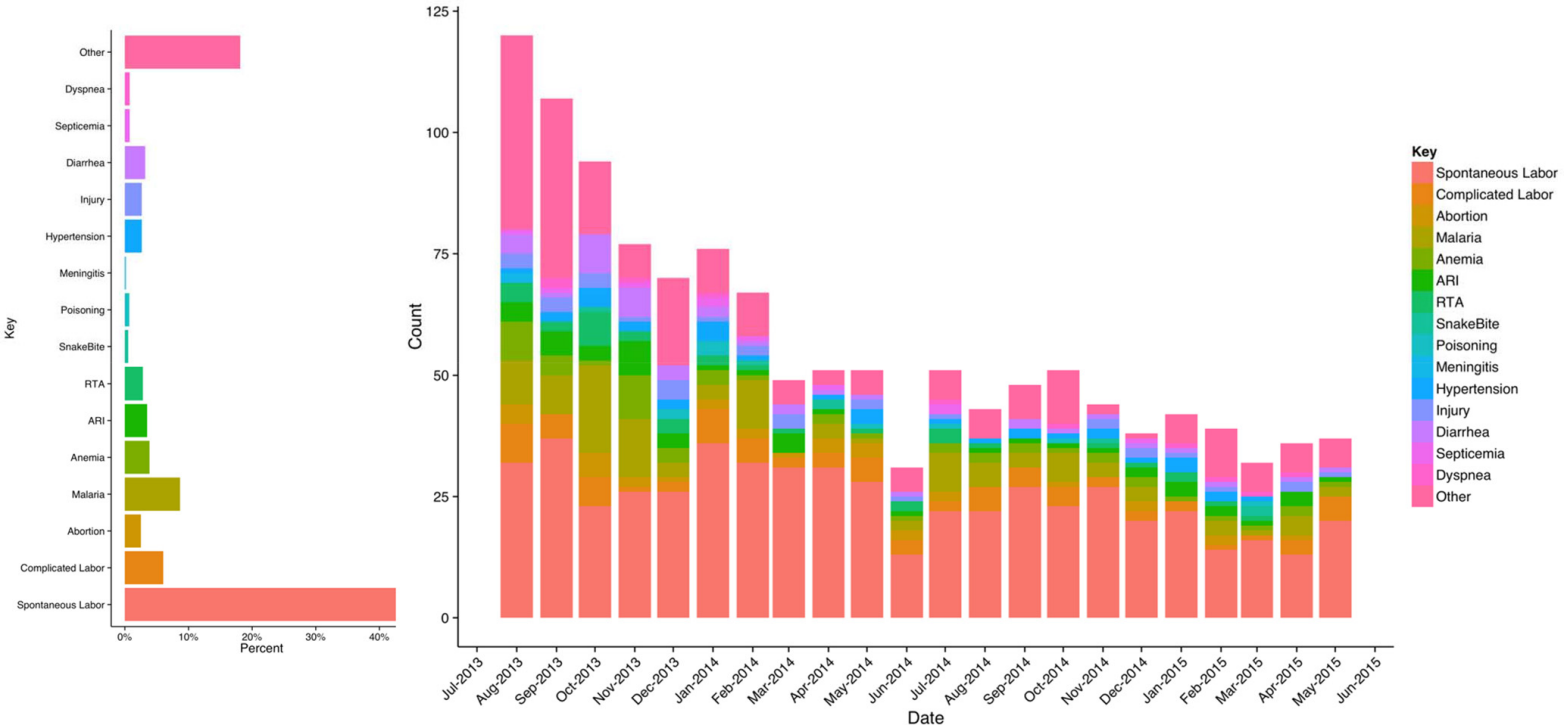
Trends in Aggregated Reasons for Referral Reported by 359 Facilities Unexposed and Exposed to the SERC System, July 2013–June 2015 Abbreviation: SERC, Sustainable Emergency Referral Care.

The referral profile changed over time as SERC progressed (Figure 2). However, regardless of time period, obstetric cases were the predominant type of referral. Consequently, nearly three quarters of patients were women. Although inappropriate use of the referral system could not be monitored directly, care for minor situations that were not emergencies tended to be labeled as “other” types of referrals. As Figure 2 shows, the proportion of such referrals declined with time, suggesting that the high initial frequency of inappropriate SERC referrals may have diminished as operations progressed.

Table 1 presents the association of SERC exposure with facility output indicators. At baseline, before the introduction of SERC services, there were approximately 53 fewer deliveries per month in hospitals in the SERC intervention area. These facilities also received fewer referrals and reported fewer upper respiratory tract infections at baseline than the facilities serving the comparison area. Cesarean delivery rates were no different between facilities in the SERC and comparison areas at baseline. SERC had no statistically significant effect on the number of deliveries; the cesarean delivery rate; the number of referrals “out” from sub-district clinics to district hospitals or the number of pneumonia cases, other respiratory tract infections, septicemia cases, or diarrheal disease cases. However, SERC did increase the number of referrals into district hospitals from CHPS workers and clinics by more than 12 patients per month and the number of accidents treated by almost 21 per month.

**FIGURE 3:**
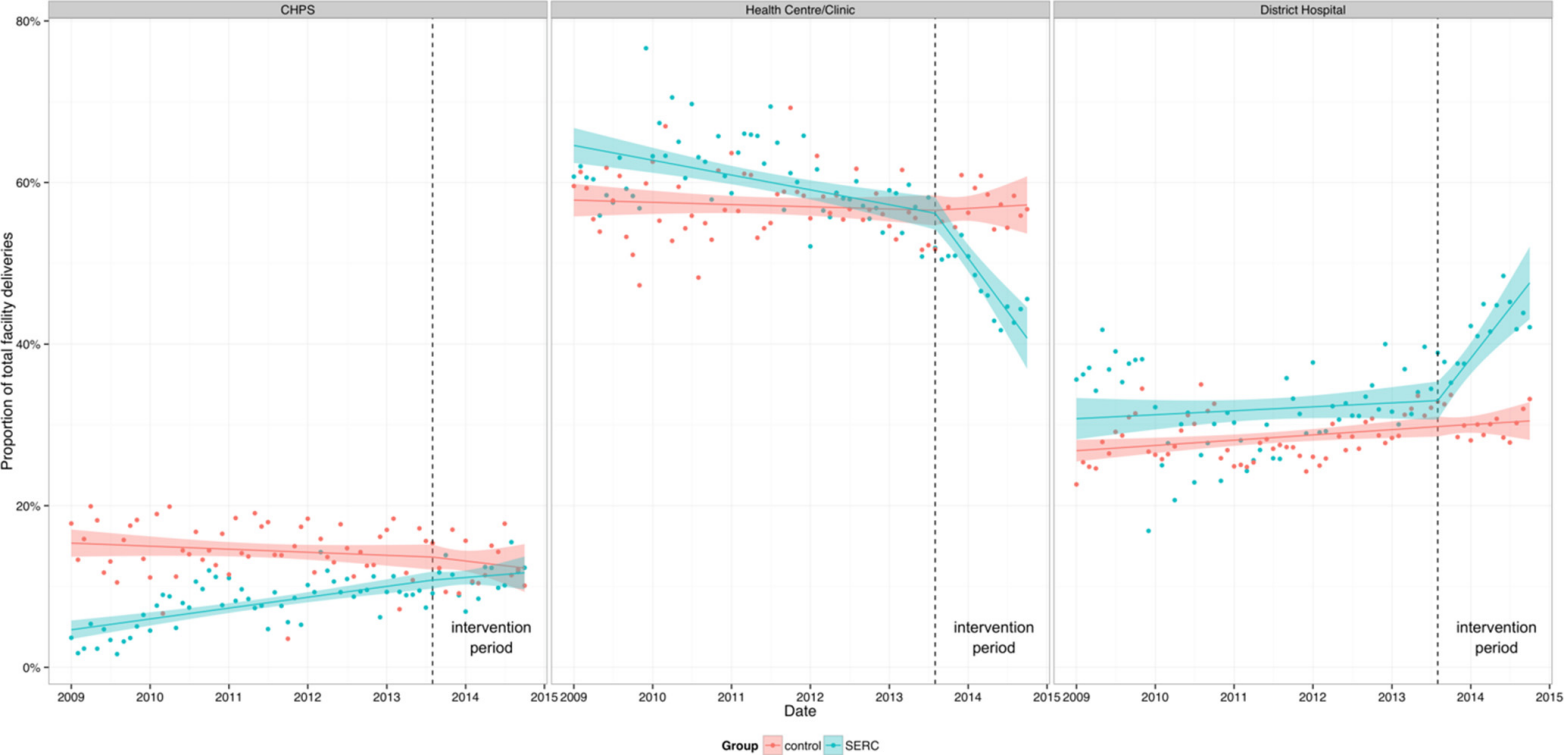
Trends in the Location of Facility Deliveries, SERC Intervention Areas vs. Comparison Areas, 2009–2015 Abbreviations: CHPS, community-based health planning and services; SERC, Sustainable Emergency Referral Care. Differences between the baseline and intervention period were statistically significant at *P*<.001 for health centers and district hospitals.

There was a shift in the location of delivery care within districts where SERC was introduced. In the SERC area, more deliveries occurred at facilities capable of acute care (i.e., district hospitals), displacing delivery care at health centers and clinics where surgical procedures are not performed (Figure 3). Hospitals staffed and equipped to provide acute care also received more referrals where SERC was operative than elsewhere (Table 1, column 3). This relocation of care was associated with a reduction in facility-based maternal mortality (incidence rate difference, −352; 95% confidence interval, −639 to −65; *P*<.05) (Table 2), although there was no significant effect on the cesarean delivery rate (Table 1, column 2). There were several specific indicators of volume of acute care episodes, and only the volume of care for accidents and for maternal emergencies appear to have been affected (Table 1, columns 3 and 8, respectively). The impact of SERC on acute care for accident victims is important, not only for the evidence shown in Table 1 but also because evidence now suggests that modest economic gains in the region have led to dramatic increases in the purchase of motorbikes, with accident-related morbidity and mortality rapidly expanding as a consequence.^18^ Time series regression results can be unstable owing to autoregressive error.^38^ Nevertheless, the relationships demonstrated in Table 1 suggest that SERC has had effects on mortality.

**TABLE 2.** Difference-in-Differences Estimates of the Impact of the SERC Initiative on Hospital-Based Maternal Mortality, Upper East and Upper West Regions, Ghana, 2009–2015

	MMR (95% CI)		Differences	
	Pre-SERC	Post-SERC	IRD (95% CI)	IRR (95% CI)
Comparison district hospitals (n = 12)	326 (272, 380)	261 (194, 328)	−65 (−140, 10)	0.80^†^ (0.61, 1.05)
Intervention district hospitals (n = 2)	618 (392, 844)	201 (22, 381)	−417** (−693, −140)	0.33* (0.13, 0.83)
**Difference-in-differences (SERC effect)**			−352* (−639, −65)	**0.41^†^ (0.15, 1.07)**

Abbreviations: CI, confidence interval; IRD, incidence rate difference (deaths per 100,000 live births); IRR, incidence rate ratio; MMR, maternal mortality ratio; SERC, Sustainable Emergency Referral Care.

Note: Estimates are from multilevel Poisson regressions of monthly hospital records of births and maternal deaths at 14 facilities in the Upper East and Upper West Regions of Ghana from 2009 to 2015. The hospital MMR is calculated as the number of facility-based deaths per 100,000 live births. The 95% confidence intervals were calculated using robust standard errors accounting for clustering at the facility level.

* *P*<.05; **P<.01; ***P<.001; ‡ *P*<.10.

Hospitals staffed and equipped to provide acute care received more referrals where SERC was operative than elsewhere.

The information monitored included process indicators such as staff compliance with protocols. Contrary to guidelines, less than half (49%) of the patients transported were accompanied by referring health workers, and receiving facilities were alerted to incoming patients in only 46% of the monitored referral episodes.

### Findings From the Health Worker Survey

Of the 110 survey respondents, over half were community health officers (56%); the remainder were clinic-based nurses (25%), midwives (13%), or physician assistants (6%). Places of work included community health posts (69%), subdistrict health centers (27%), and hospitals (4%). Nearly three-quarters (74%) of the respondents had personally referred patients using SERC's transport service since the program launched.

#### Perceived Effectiveness and Safety of Motorking Ambulances

The majority of health workers (66%) considered SERC to be “very effective” in improving the community- and subdistrict-level emergency referral system, and 33% considered Motorkings to be “somewhat effective.” Most health workers perceived the Motorking to be safe, with 26% categorizing the vehicle as “very safe” and 61% considering it to be “somewhat safe.”

Two-thirds of health workers considered SERC to be “very effective” in improving the emergency referral system.

#### Driver Dedication and Availability

Respondents generally perceived drivers to be dedicated to their roles (56% reported finding them “very dedicated”; 41% found them “somewhat dedicated,” and only 3% found them “not at all dedicated”). However, there were instances when health workers were unable to promptly contact the volunteer drivers. When asked whether health workers at ambulance stations should be trained to drive Motorkings in such instances, the majority of respondents (75%) said yes.

#### Protocol Adherence

Protocol noncompliance was evident for some aspects of care. For instance, referring facilities often failed to call in advance to alert receiving facilities of an incoming patient. Moreover, many patients transferred were unaccompanied by a health worker despite the protocol requiring it. Although surveyed health workers nearly universally affirmed the importance of these procedures, 67% of the referred patients who were interviewed reported that they had been transferred without accompaniment. When workers were asked why they were unable to accompany the patient, the most commonly cited reasons (respondents could choose more than one) were that another health worker accompanied the patient (37%); the respondent was the only staff member at the facility and could not leave the post (35%); or the respondent was attending another patient (32%). Some respondents reported that they did not accompany the patient because they did not feel comfortable riding in the ambulance (16%), while 4% thought the patient would not benefit from riding with a health worker. Protocols also obligate receiving facilities to provide patient outcome feedback to the referring facilities for every case, but this requirement was typically ignored.

Referring facilities often failed to alert receiving facilities of an incoming patient, and many patients were unaccompanied by a health worker during transit.

#### Frontline Worker Perspectives

Surveyed health workers were asked to identify the primary challenges to effective emergency referral services (the health workers could choose more than one challenge). Poor road conditions (95%) was the most commonly reported challenge, followed by lack of driver motivation (59%); cultural practices that delay care seeking and lack of knowledge of the importance of seeking care (40%); poor communications networks (32%); and adverse weather conditions (29%). Less frequently reported responses included patient inability to take time away from work or family obligations (20%); the cost or unavailability of fuel (19%); poor communication between health facilities (18%); lack of readily available transport options (13%); or lack of Motorking acceptability (12%).

### Findings From the Qualitative Systems Appraisal

The qualitative systems appraisal shed light on the acceptability of services and on community perspectives on potential areas for improvement. Four main categories of themes emerged during data analysis: community endorsement of SERC; logistical challenges; communication challenges; and interpersonal relationships.

#### Community Endorsement of SERC

Overall, community members strongly endorsed SERC and expressed appreciation for the service. SERC was generally perceived as reliable and reactive, with a committed staff that supported the system. For instance, a woman who had used SERC said this:

It sent me to the clinic to deliver and I did that safely without any bad thing happening to me. I delivered safely. That is the beauty of it.

Several users reported that they would recommend SERC services to anyone in need of emergency care. The removal of fees for pregnant women and children under 5 was seen as a key contributor to high SERC uptake. Although some participants preferred 4-wheeled ambulances, respondents generally believed that the 3-wheeled ambulance was better than the available alternatives, such as walking, bicycles, donkey carts, or motorbikes:

The removal of fees for transporting pregnant women and children under 5 was seen as a key contributor to high uptake.

It has been so beneficial to the pregnant women and the children under 5 because they do not pay when the vehicle is transporting them. In the past, we used to transport pregnant women in donkey carts and on bicycles but today there is ready and reliable means of transport for them in emergencies. —Community volunteer participating in an FGD

Some participants acknowledged that 3-wheeled vehicles such as the Motorking can traverse narrow passages that are inaccessible to 4-wheeled vehicles:

If not for the Motorking, women, especially pregnant women and children, would have been suffering a lot. … It is able to go to the interior [of communities] to carry cases like the one I told you about with the woman who was in labor and nearly died if not for the sake of the Motorking ambulance. —Community subchief, in an IDI

Perceptions of reduced delays and increased numbers of facility-based births as a result of SERC were mentioned by several participants, along with the impression that SERC services were helpful, safe, quick, and lifesaving:

When a woman is in labor and is not quickly sent to the health facility, she might deliver. She might also lose either the baby or even herself. Kids like this, once they are weak, they can easily pass on. So the impact I see is that the emergency referral saves lives. —SERC driver participating in an FGD

Community and household consensus endorsing SERC was uniformly evident in each FGD and IDI and was a key determinant of the sustainability of the system.

#### Logistical Challenges

While communities were receptive to SERC services, several logistical challenges were identified. Some intervention areas remained inaccessible due to harsh weather and terrain, especially during the rainy season. (These challenges were perceived to affect all vehicle types, not just the Motorkings.) Some communities conveyed interest in overcoming logistical or geographical barriers through collective action or political advocacy. As 2 FGD respondents suggested:

Some communities conveyed interest in overcoming logistical or geographical barriers through collective action or political advocacy.

I am of the view that the community members can contribute something, however little, and seek assistance from the authorities to work on our routes or roads for us.

Our youth, if they could help us to repair our roads small, small [bit by bit], and when the motors come, they can be running without problems.

Although concerns about roads did not constrain SERC use, some participants noted that communities that were remote from an ambulance station anticipated delay and often sought alternative means of emergency care. Indeed, this observation is consistent with GIS data analyses showing that communities located far from ambulance stations had lower use rates than proximate communities.

#### Communication Challenges

Communication problems introduced further complications. Poor phone networks, which are common in rural Ghana, exacerbated service delays. Although this did not compromise care seeking resolve, solutions to communication gaps sometimes involved walking great distances to alert a health worker or volunteer.

Poor phone networks did not compromise decisions to seek care, but solutions sometimes involved walking great distances to alert a health worker or volunteer.

Patient perspectives on comfort during transport varied: Some patients described the vehicle as being unstable and uncomfortable, while others described feeling very safe, with minimal discomfort. This problem was associated with poor road quality. Any discomfort, however, did not appear to be severe enough to deter people from using SERC in the event of emergencies:

There are issues like discomfort, safety, and others when you are being transported, but as a sick person you do not have those issues in mind when there is an emergency. Anything that can hurriedly get you to the place on time is what you will be looking for. All vehicles have the tendency of falling when transporting people so it will not be fair relating safety issues to the Motorking alone. —Man participating in an FGD

Everyone has his problem, and when the vehicle picked me [up] the driver knew that it was a painful thing being in labor, so they also became careful with the way they were driving and we got there safely. Now I will not be able to speak for another person, but for my experience it was comfortable. —Woman who had used SERC, in an IDI

Community members also expressed support for improving the work conditions for drivers. There were concerns over drivers being exposed to unfavorable weather and the risk of robbery during late-night service. Although no such incidents were reported, a few drivers worried about driving at night:

There are beasts at night and also ghosts. From where I come, there are so many spirits that it is not advisable to move out at night. The people sit protected in the vehicle while you are left alone in front. In addition to that you are not supposed to speed the vehicle, and you can imagine how exposed you are if someone intends to harm you. —SERC driver participating in an FGD

Drivers advised SERC to develop roadside repair protocols for addressing unanticipated mechanical problems. Drivers also noted that personal transportation was a challenge, as many drivers had no means of personal transportation home following late-night referrals. Drivers were provided with 2 bars of soap monthly as a token of appreciation, and this was universally perceived as being insufficient. Cash incentives were preferred by all drivers who were interviewed. Staff participating in an FGD considered cash payments as being critical to sustaining driver motivation in the future. Some community members recommended that SERC choose drivers from the ranks of existing community health volunteers, given prevailing volunteer commitment to community health.

#### Community Trust and Expectations

Some participants noted instances of mistrust between health workers and drivers. For example, a driver mentioned an episode where the network was down but a health worker accused him of having turned off his phone. In another example, a man explained during an FGD how at times users might misconstrue basic triage practices as health worker neglect:

Some of the pregnant women will be complaining that they came and they are thrown away, they don't care about them. Because there is no understanding between the pregnant women and the midwife when she tells them it's not time for them to deliver and they should wait. Because of that, the women say the workers are not serious, but for me, the way I know about the work, I know they are serious.

Although some patients experienced negative interactions with health workers, many described satisfaction with their performance during emergencies:

We think that the child was saved by the nurses because of the timeliness of our arrival. We were happy when we got into the hands of the nurses. —Woman who had used SERC, in an IDI

Drivers expressed concern that the community lacked respect for their contribution. Some community members believed that drivers were paid employees rather than volunteers, and some drivers reported frustration over receiving dismissive and ungrateful comments. Although most community members interviewed indicated gratitude for drivers' services, some complained that drivers operated Motorkings at unsafe speeds.

## DISCUSSION

Mixed-methods implementation research enriched learning about the scalability, acceptability, and potential impact of implementing a community-based emergency referral system in a severely resource-constrained setting. The findings suggest that the strategies used for the emergency referral system can be adapted to the needs of impoverished, remote communities elsewhere in Ghana.

Overall, the SERC system was well received by communities and health workers alike as an effective means of reducing acute care risks. A key lesson learned was the importance of people-centered planning for obtaining and sustaining community endorsement and use of services. Without the engagement of community leaders from the very beginning, acceptance of the program would have been limited. Focused outreach targeting heads of household and familial gatekeepers is also crucial to ensuring continued support and understanding of services. Moreover, the collaborative role of transportation authorities and vehicle manufacturers in the planning, training, and implementation processes proved vital to program success.

While SERC aimed to use process evaluation results to improve system functioning, the pursuit of such improvements was constrained by resource limitations, poor communication network infrastructure, and impassible roads. Nearly all the health workers consulted in this appraisal expressed willingness to use emergency radios to offset poor mobile phone coverage. However, given limited funds for equipment purchases and lack of locally available communication equipment, use of radio devices could not be implemented. Instead, workers were obligated to develop improvised solutions when networks were not functioning.

The Motorking was locally available, affordable, and suitable for traversing rough terrain. Nonetheless, Motorking ambulances received mixed reviews for comfort and safety, indicating a need to explore additional equipment options. A costing analysis that compares 3-wheeled motorcycle ambulances with enhanced Motorkings or higher-quality vehicles is warranted. Similarly, strategies should be investigated for determining an appropriate and sustainable incentive and recruitment system for drivers in order to minimize turnover, improve motivation, and optimize efficiency.

Quite apart from equipment considerations, the quality of emergency care services will be limited by the poor state of infrastructure more generally. Several of the community members who were interviewed expressed concern about the status of the development of primary health care facilities and the slow pace of CHPS implementation, highlighting the fact that effective referral care requires a fully functioning primary health care system.

Effective referral care requires a fully functioning primary health care system.

The SERC experience attests to the value of routine monitoring and evidence-based supervision, in conjunction with refresher training for health workers and volunteers. Lack of accountability mechanisms, supervision, and training can lead to poor adherence to protocols.

Feedback mechanisms are needed to foster timely implementation of systems improvements. For instance, after it was discovered that 30% of the trip report forms were incomplete, the forms were simplified, the format of review meetings was revised, and GIS-based vehicle tracking procedures were instituted to facilitate practical use of data for decision making. Similarly, adhering to a routine vehicle maintenance protocol that ensures prompt repairs was found to be crucial for preventing breakdowns and minimizing service disruptions.

Training for quality assurance is important. Although most patients reported positive experiences with staff involved in facilitating referrals, some patients experienced negative or insensitive comments. While clinical skills are crucial to operations, it is equally essential to foster health workers' patience and understanding of patients' perspectives on the quality of emergency care operations.

For quality emergency care, clinical skills, patience, and understanding of patients' perspectives are equally essential.

Some volunteer drivers perceived community members to be unappreciative of their services. Although FGD participants may have been reluctant to criticize drivers, the general discussion suggests that drivers were, in fact, appreciated, and participants generally agreed that the incentives provided to drivers should be increased. The exchanges among FGD participants nonetheless suggested a need for durbars and other means of community engagement that would promote awareness of the lifesaving service and dedication of volunteers.

Focus group discussions suggested a need for community engagement to promote awareness of the service and dedication of volunteers.

Remote communities sometimes preferred to find their own means of transport to offset ambulance delays. This fact attests to the need for implementation research that investigates the mechanisms such communities use for emergency referral and transport. Community-based solutions to referral problems may be relevant to operations more generally.

SERC has made its impact by transporting emergency cases, at considerable cost, to distant hospitals where physicians are available to provide essential acute care. However, bypassing subdistrict clinics and relocating care to hospitals is less sustainable than developing service capability at the subdistrict level. While bypassing for delivery is a logical and common strategy in Ghana^40^^,^^41^ and elsewhere in Africa,^42^^,^^43^ the implication of this finding is clear: A new round of implementation research is needed to explore implementing SERC in concert with a program that trains midlevel providers to manage emergencies directly.

## CONCLUSION

While facility-based emergency health care is important to reducing mortality, facility-focused approaches can fail to achieve their full lifesaving potential in the absence of effective referral. Moreover, if receiving facilities are poorly equipped, inadequately staffed, and unable to respond to clinical emergency needs, effective referral will be little more than a program for relocating mortality. The SERC time series research presented in this article attests to the lifesaving potential of redirecting referral to facilities where emergencies can be competently managed. In the areas where SERC rechanneled acute care to specified facilities, we found decreased facility-based maternal mortality and accident-related deaths relative to comparison facilities. In the future, SERC could expand its intervention regimen with training and capacity building to enable more frontline care providers in smaller facilities to more effectively manage emergencies that arise. This strategy would offset the existing strategies for bypassing substandard care facilities.

In the future, SERC could train more frontline care providers to more effectively manage emergencies that arise.

Just as SERC's success was dependent on implementation research and evidence, effective scale-up of these results will require effective systems approaches to replication trials in other regions of Ghana, with contrasting social, economic, and ecological conditions. The need for reform of referral systems persists throughout the country, but care should be taken to develop solutions that are informed by SERC, yet tailored to local contexts in the central and southern regions. The transition from pilot to trial clarified training and engagement requirements; replication of SERC elsewhere in Ghana could clarify the practical milestones in establishing a large-scale system of referral care.

The Ghana Health Service has adopted the SERC model and has included it in the national CHPS implementation guidelines. Donors including the Japan International Cooperation Agency, the Korean International Cooperation Agency, and the U.S. Agency for International Development have funded the purchase of Motorkings, which are being used in 4 of Ghana's 10 regions, with plans to use them in 2 additional regions.

## Notes

**Peer Reviewed**
